# Supplementary material for: Content-rich biological network constructed by mining PubMed abstracts
Source: BMC Bioinformatics. 2004 Oct 8;5:147. doi: 10.1186/1471-2105-5-147 (PMC528731; doi:10.1186/1471-2105-5-147)
Supplement: Additional File 2 — The original results of the above study (non-essential files are deleted to keep the file size under the limit set by BMC bioinformatics). [file 1471-2105-5-147-S2.bz2 › chilibotAdditionalFile2/dip40/693ID11994151E18018/html/TOLA_TOLB.html]

 


 **TOLA** and **TOLB** 
  
Found 36 abstracts in PubMed, retrieved 36.  
 

 What does Google say? 
 PDF only 
| .edu only 

---

**Interactive relationship** (e.g. stimulation, inhibition, etc)

**Neutral relationship**- Mutational analysis of the  **TolA**  C terminal domain of Escherichia coli and genetic evidence for an interaction between  **TolA**  and  **TolB** .  Ref: 12142433 J Bacteriol, 2002
- The  **TolA**   **TolB**  interaction was confirmed by cross linking experiments on purified proteins.  Ref: 11994151 Mol Microbiol, 2002
- These results demonstrate that the  **TolA**   **TolB**  interaction allows the formation of a trans envelope complex that brings the inner and outer membranes in close proximity.  Ref: 11994151 Mol Microbiol, 2002
- Moreover, we show that the interaction between  **TolA**  and  **TolB**  is required for the uptake of colicin A and for the membrane integrity.  Ref: 11994151 Mol Microbiol, 2002
- Distinct regions of the colicin A translocation domain are involved in the interaction with  **TolA**  and  **TolB**  proteins upon import into Escherichia coli.  Ref: 9466263 Mol Microbiol, 1998
- **TolB**  dimerizes and the periplasmic domain of  **TolA**  interacts with YbgF and  **TolB** .  Ref: 11994151 Mol Microbiol, 2002
- This region and the other regions responsible for the interaction with  **TolA**  and  **TolB**  have been mapped precisely within the colicin A N terminal domain and appear to be arranged linearly in the colicin sequence.  Ref: 11703658 Mol Microbiol, 2001
- The Tol Pal system function requires an interaction between the C terminal domain of  **TolA**  and the N terminal domain of  **TolB** .  Ref: 11994151 Mol Microbiol, 2002
- Our results indicate that the central domain of  **TolA**  TolAII is sufficient to interact with YbgF, that the C terminal domain of  **TolA**  TolAIII is sufficient to interact with  **TolB** ,.  Ref: 11994151 Mol Microbiol, 2002
- These results suggest that the interactions of  **TolA**  and  **TolB**  with porins might take place in vivo and might be concomitant events participating in porin assembly.  Ref: 9393690 J Bacteriol, 1997
- Transmembrane domains of TolQ, TolR and  **TolA**  interact in the cytoplasmic membrane, while  **TolB**  and Pal form a complex near the outer membrane.  Ref: 9701827 Mol Microbiol, 1998
- Transmembrane domains of TolQ, TolR, and  **TolA**  interact in the cytoplasmic membrane, while  **TolB**  and Pal form a complex near the outer membrane.  Ref: 9851983 J Bacteriol, 1998
- The isolation of suppressor mutants of  **tolA**  mutations in the  **tolB**  gene confirmed an interaction between TolAIII and the N terminal domain of  **TolB** .  Ref: 12142433 J Bacteriol, 2002

**Non-interactive relationship** (e.g. studied together, co-existance, homology, etc.)

- The nucleotide sequence of a 2.8 kilobase fragment containing the  **tolA**  and  **tolB**  genes was determined.  Ref: 2687247 J Bacteriol, 1989
- Bacteriocin sensitivity tests performed on a collection of E. coli envelope mutants, and isolation and characterization of E. coli bacteriocin 28b insensitive mutants, showed that the core lipopolysaccharide, outer membrane proteins OmpA and OmpF, and TolQ,  **TolA** , and  **TolB**  proteins are involved in bacteriocin 28b lethal activity.  Ref: 8595593 Can J Microbiol, 1996
- The colicin A N terminal domain was also shown to form a trimeric complex with  **TolA**  and  **TolB** .  Ref: 9466263 Mol Microbiol, 1998
- The N terminal domains of colicins A and E1 have been shown to interact with  **TolA** , and the N terminal domain of colicin E3 has been shown to interact with  **TolB** .  Ref: 9466263 Mol Microbiol, 1998
- Mutants of Escherichia coli K12 carrying exc mutations inducing the release of the plasmid pBR322 encoded beta lactamase EC 3.5.2.6 into the extracellular medium were analysed and compared with previously described excretory mutants carrying lky mutations associated with the release of alkaline phosphatase and to  **tolA**  and  **tolB**  mutants, originally described as tolerant towards various colicins.  Ref: 3312962 Mol Gen Genet, 1987
- **TolA** , TolQ, and TolR are localized in the inner membrane while  **TolB**  is periplasmic, although a small amount of membrane associated  **TolB**  is always found.  Ref: 7744737 J Biol Chem, 1995
- Strains of Escherichia coli K12 carrying a  **tolA** ,  **tolB** , lky or exc mutation located at min 16.5 on the genetic map released periplasmic proteins into the extracellular medium.  Ref: 2573819 Mol Gen Genet, 1989
- The  **TolA** ,  **TolB** , TolQ, and TolR proteins are involved in maintaining the integrity of the Escherichia coli outer membrane and in the import of group A colicins and filamentous phage DNA.  Ref: 7744737 J Biol Chem, 1995
- This cluster was found to consist of seven genes in the order orf1 tolQ tolR  **tolA**   **tolB**  oprL orf2.  Ref: 10735848 J Bacteriol, 2000
- Mutations in fii or  **tolA**  of the fii  **tolA**   **tolB**  gene cluster at 17 min on the Escherichia coli map render cells tolerant to high concentrations of the E colicins and do not allow the DNA of infecting single stranded filamentous bacteriophages to enter the bacterial cytoplasm.  Ref: 3294803 J Bacteriol, 1987
- We consider a variety of different colicins, including A, B, D, E1, E3, Ia, M and N, that penetrate through the porins OmpF, FepA, BtuB, Cir and FhuA, to subsequently interact with a few targets in the periplasm, including  **TolA** ,  **TolB** , TolC and TonB.  Ref: 12423783 Biochimie< MedlineTA>Biochimie, 1987
- Mutations in tolQ, tolR,  **tolA** ,  **tolB** , or pal genes result in sensitivity to bile salts and the leakage of periplasmic proteins.  Ref: 10633120 J Bacteriol, 2000
- **TolB**  and the central domain of  **TolA**  interact in vitro with the outer membrane porins.  Ref: 9701827 Mol Microbiol, 1998
- In the present study we cloned and mutagenized the orf1, tolQ, tolR,  **tolA** , and  **tolB**  genes from P. putida KT2440, which were located upstream of the oprL gene.  Ref: 10940016 J Bacteriol, 2000
- It is organized into two complexes, one near the outer membrane between  **TolB**  and PAL and one in the cytoplasmic membrane between  **TolA** , TolQ, and TolR.  Ref: 10419942 J Bacteriol, 1999
- The Tol Pal system of Escherichia coli is composed of the YbgC, TolQ,  **TolA** , TolR,  **TolB** , Pal and YbgF proteins.  Ref: 11994151 Mol Microbiol, 2002
- Group A colicins need proteins of the Escherichia coli envelope Tol complex  **TolA** ,  **TolB** , TolQ and TolR to reach their cellular target.  Ref: 9466263 Mol Microbiol, 1998
- The interactions of colicin A N terminal domain deletion mutants with  **TolA**  and  **TolB**  were investigated.  Ref: 9466263 Mol Microbiol, 1998
- The deduced amino acid aa sequence of the H. influenzae TolQ, TolR,  **TolA**  and  **TolB**  show 6 1 and 62% identity with Escherichia coli TolQRAB proteins, respectively.  Ref: 8921895 Gene, 1996
- In this study, we demonstrate that the genes tolQ, tolR, and  **tolA** , but not  **tolB** , tolC, and ompF, are required for the internalization of cloacin DF13 and they are not involved in the transport of ferric aerobactin.  Ref: 8419302 J Bacteriol, 1993
- Uptake of the mutated colicin U DTMVV depended on TonB and ExbB and still remained  **TolA**  and TolQ dependent, but no longer required  **TolB** .  Ref: 9682486 FEMS Microbiol Lett, 1998
- **TolA** , TolQ, and TolR are inner membrane proteins,  **TolB**  is a periplasmic protein, and Pal, the peptidoglycan associated lipoprotein, is anchored to the outer membrane.  Ref: 11790745 J Bacteriol, 2002
- The three genes  **tolA** ,  **tolB** , and fii are shown to reside on a 4.3 kilobase fragment of the Escherichia coli chromosome.  Ref: 3001021 J Bacteriol, 1986
- Subcloning experiments and complementation studies showed that lky and exc mutations were located either in the previously described  **tolA**  and  **tolB**  genes or in the newly characterized excC and excD genes.  Ref: 2573819 Mol Gen Genet, 1989
- Analysis of polar mutations, reverse transcriptase PCR assays, and transcriptional fusion constructs with a promoterless lacZ gene revealed that the genes are arranged in two operons orf1 tolQ tolR  **tolA**   **tolB**  and oprL orf2.  Ref: 12486055 J Bacteriol, 2003
- The Tol Pal system of the Escherichia coli envelope is formed from the inner membrane TolQ, TolR and  **TolA**  proteins, the periplasmic  **TolB**  protein and the outer membrane Pal lipoprotein.  Ref: 11115123 Mol Microbiol, 2000
- Our results demonstrate that different regions of the colicin A N terminal domain interact with  **TolA**  and  **TolB** .  Ref: 9466263 Mol Microbiol, 1998
- fii, a bacterial locus required for filamentous phage infection and its relation to colicin tolerant  **tolA**  and  **tolB** .  Ref: 3001021 J Bacteriol, 1986
- They first interact with receptors located at the surface of the outer membrane and are then transferred across the cell envelope in a process that requires energy and depends upon accessory proteins  **TolA** ,  **TolB** , TolC, TolQ, TolR which might play a role similar to that of the secretory apparatus of eukaryotic and prokaryotic cells.  Ref: 3139035 Biochim Biophys Acta, 1988
- Colicin A interacts with  **TolA**  and  **TolB**  via distinct regions of its N terminal domain.  Ref: 11703658 Mol Microbiol, 2001
- In the presence of the purified central domain of  **TolA**  TolAIIHis, the  **TolB**  porin complexes disappeared to form TolAIIHis porin complexes.  Ref: 9393690 J Bacteriol, 1997
- Polar and nonpolar mutations of the P. putida tolQ, tolR,  **tolA** , and  **tolB**  genes were generated in vitro by using the omega Km r interposon, which carries two transcriptional stop signals, or a promoterless xylE cassette, lacking any transcriptional stop signal, respectively.  Ref: 10940016 J Bacteriol, 2000
- The TolQ, TolR,  **TolA** ,  **TolB** , and Pal proteins appear to function in maintaining the integrity of the outer membrane, as well as facilitating the uptake of the group A colicins and the DNA of the infecting filamentous bacteriophages.  Ref: 8763928 J Bacteriol, 1996
- The TolQ TolR  **TolA**  proteins form a complex in the cytoplasmic membrane, while  **TolB**  and Pal interact near the outer membrane.  Ref: 10474183 FEMS Microbiol Lett, 1999
- In Pseudomonas putida, the seven genes, orf1, tolQ, tolR,  **tolA** ,  **tolB** , oprL, and orf2, which encode the proteins of this complex, are clustered in a 5.8 kb region of chromosomal DNA.  Ref: 12486055 J Bacteriol, 2003
- **TolA**  and TolR are anchored to the inner membrane by a single transmembrane domain, TolQ is an integral membrane protein with three transmembrane segments, and  **TolB**  has recently been found to be periplasmic although it is partially membrane associated.  Ref: 7744736 J Biol Chem, 1995
- Mutants of E. coli K 12  **tolA**  and  **tolB**  which leak periplasmic proteins mimic excretion and release the haemolysin into the growth medium.  Ref: 3900640 Mol Gen Genet, 1985
- Nucleotide sequences of the  **tolA**  and  **tolB**  genes and localization of their products, components of a multistep translocation system in Escherichia coli.  Ref: 2687247 J Bacteriol, 1989
- A genetic analysis presented in this paper showed that some exc and lky mutations belonged to the  **tolA**  and  **tolB**  complementation groups.  Ref: 3312962 Mol Gen Genet, 1987
- **tolA** ,  **tolB**  and excC, three cistrons involved in the control of pleiotropic release of periplasmic proteins by Escherichia coli K12.  Ref: 3312962 Mol Gen Genet, 1987
- Most of the amino acid residues of  **TolA** ,  **TolB** , TolR and Pal are localized in the periplasm.  Ref: 10474183 FEMS Microbiol Lett, 1999
- Data from cloning, Tn5 mutagenesis, and P1 transduction studies are consistent with the gene order sucA fii  **tolA**   **tolB**  aroG near 17 min on the E. coli map.  Ref: 3001021 J Bacteriol, 1986
- Analysis of double mutants strains carrying mutation staA 2 and a  **tolA** ,  **tolB** , excC or excD periplasmic leaky mutation showed that staA suppression was allele specific which suggested that proteins  **TolA**  and StaA might directly interact.  Ref: 2040438 FEMS Microbiol Lett, 1991
- Isolation and characterization of the Haemophilus influenzae tolQ, tolR,  **tolA**  and  **tolB**  genes.  Ref: 8921895 Gene, 1996
- Sequence data showed that these genes are clustered in a 6 kb segment of DNA with the gene order orf1 tolQ tolR  **tolA**   **tolB**  pal orf2 a newly identified open reading frame encoding a 29 kD9 protein.  Ref: 8763928 J Bacteriol, 1996
- Furthermore, bacteriocin 28b translocation requires proteins  **TolA** ,  **TolB** , and TolQ.  Ref: 8595593 Can J Microbiol, 1996
- The predicted amino acid sequences of V. cholerae TolQ, TolR,  **TolA** , and  **TolB**  showed significant similarity to the corresponding E. coli sequences.  Ref: 10692381 J Bacteriol, 2000
